# Cost-Effectiveness Analysis of 23-Valent Pneumococcal Polysaccharide Vaccine Program for the Elderly Aged 60 Years or Older in Shanghai, China

**DOI:** 10.3389/fpubh.2021.647725

**Published:** 2021-05-24

**Authors:** Xiaodong Sun, Yuekun Tang, Xiaoying Ma, Xiang Guo, Zhuoying Huang, Jia Ren, Jing Qiu, Hongli Jiang, Yihan Lu

**Affiliations:** ^1^Shanghai Municipal Center for Disease Control and Prevention, Shanghai, China; ^2^School of Public Health, Fudan University, Shanghai, China

**Keywords:** cost-effectiveness, the elderly, PPSV-23, Shanghai, vaccine

## Abstract

**Background:** The pneumococcal vaccine has been considered as the most effective measure to prevent pneumococcal diseases. In 2013, Shanghai launched a major public health program to vaccinate people aged 60 years or older with 23-Valent Pneumococcal Polysaccharide Vaccine (PPSV-23) free of charge. By the end of June 2020, a total of 1.56 million old people had been vaccinated free of charge.

**Objective:** To evaluate the cost-effectiveness of PPSV-23 vaccination program in Shanghai from the health system perspective.

**Methods:** According to the actual number of people aged 60 years or older with PPSV-23 vaccination in Shanghai from 2013 to 2018, a multi-cohort Markov model for life-time was developed to compare health and economic outcomes of vaccinated people vs. if they were not vaccinated for PPSV-23. Cost effectiveness was reported as incremental cost effectiveness ratio (ICER). A 5% discount rate was used for both costs and health outcomes. In addition, one-way sensitivity analysis was used to test the model's robustness.

**Results:** By the end of 2018, a total of 1,091,967 people aged 60 years or older were vaccinated with PPSV-23 in Shanghai, China. Comparing with the unvaccinated circumstances, PPSV-23 vaccination would cost US $19.62 million more and receive an additional 10,321.3 quality-adjusted life-year (QALY). PPSV-23 was associated with the ICER of $190.1 per QALY gained. The Results were sensitive to the variation of vaccine effectiveness against community-acquired pneumonia (CAP), and disease incidence, mortality, and costs of CAP. In all sensitivity analysis, the PPSV-23 was economical.

**Conclusion:** The PPSV-23 vaccination program in Shanghai was cost-effective. With the further development of the project, the administrative costs of the vaccine will be reduced, making it more cost-effective.

## Introduction

*Streptococcus pneumoniae* is one of the major causes of pneumonia, bacteremia, meningitis, and has high morbidity and mortality, especially in the elderly. According to Sheng-fan et al. (1), a total of 27,723 hospitalized patients with pneumonia were reported in Shanghai in 2011, of which the number of people over 65 years old was the most, accounting for 44.65% of the total. At the same time, the average hospitalization time of people over 65 years old was 16.11 days, which was higher than that of 13 days in the total population. The average hospitalization cost for people over 65 years old was 14,520 yuan (1 Chinese yuan = 0.1521 US dollar in 2013), which was also the highest among that of all age groups. It certainly caused huge health and economic burden.

The World Health Organization points out that vaccine is the most effective preventive measure for pneumococcal diseases (2). The United States (3, 4), Britain (5), Europe (6) have included pneumococcal vaccine in their immunization programs or recommended vaccination guidelines, and put forward different vaccination recommendations for different age groups. Studies in the United States, Britain, Germany, and other countries have confirmed that vaccination can effectively prevent the occurrence of pneumococcal diseases and reduce deaths (7–9).

PPSV-23 was licensed for use in China in 1998 (10), including 6B, 14, 19F, 19A, 23F and other common serotypes of pneumoniae in China (2). Model-based studies with some local parameters from district-based surveys in Shanghai show that the use of pneumococcal vaccination is an effective strategy to prevent pneumococcal diseases (11). Shanghai has implemented a major public health service project to provide people 60 years and older with a free dose of PPSV-23 since 2013, financed by the municipal government. By the end of June 2020, a total of 1.56 million older people had been vaccinated free of charge. The vaccination coverage rate is estimated at 30% of the target population. The municipal government of Shanghai invested 177 million yuan, and the sixteen district governments of Shanghai invested an additional 23 million yuan in purchasing the vaccine and promoting the project. To better evaluate the effectiveness of the project, Shanghai Municipal Center for Disease Control and Prevention (SCDC) conducted a cohort study in the same period in 2013 to select 1,214 vaccinated elderly and 2,387 unvaccinated elderly for a follow-up to explore the effect of PPSV-23 vaccination (12). Taking the major public health service project in Shanghai and the fact that antibiotics effectively treat pneumococcal diseases into account, our study aims to investigate the significance of state-funded free vaccination of PPSV-23 in the elderly from the perspective of economic evaluation.

## Materials and Methods

### Model Structure and Target Population

A Markov model design was selected after a review of the existing cost-effectiveness literature (13–15) and consultation with experts. The model tracked eligible cohorts until death or 100 years of age. The target population was people who vaccinated PPSV-23 from 2013 to 2018 in the major public health program in Shanghai.

**Figure 1:**
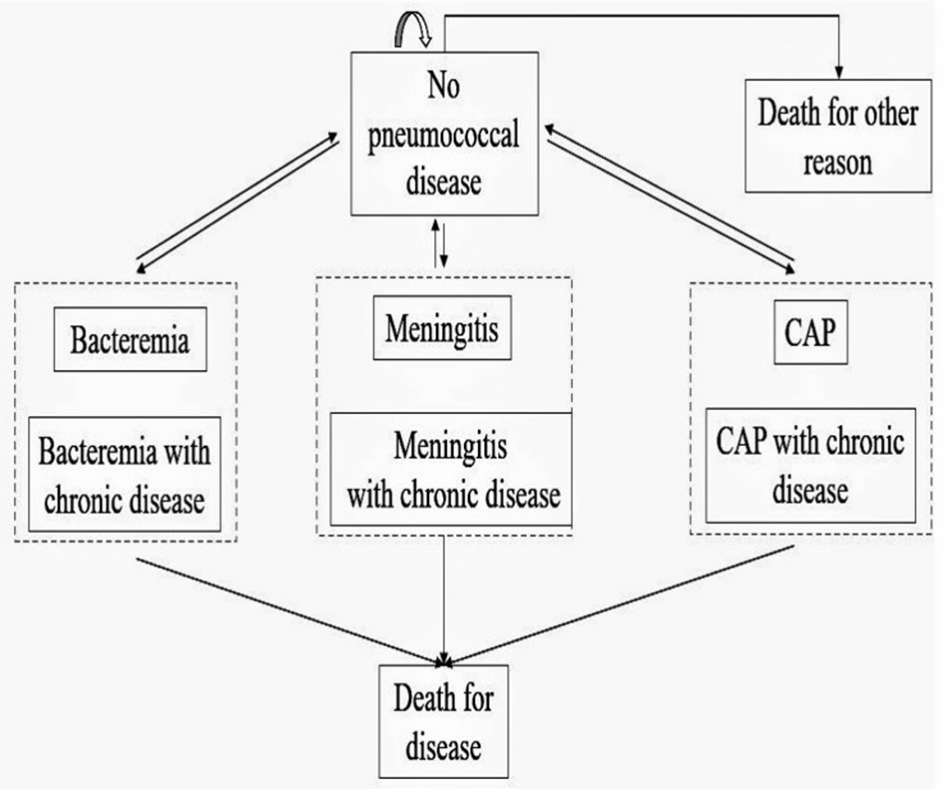
Model structure.

The model assumed that vaccinated and unvaccinated individuals would experience each of six different health events: no pneumococcal diseases, bacteremia, meningitis, community-acquired pneumonia, death for pneumococcal diseases, and death from all other causes. Previous studies have shown that patients with chronic diseases have more serious symptoms than those without chronic diseases after being infected with pneumococcal diseases (16). Therefore, each disease infection state was divided into pneumococcal disease infection and pneumococcal disease infection complicated with chronic disease, and we classified individuals with chronic diseases as high-risk population. The specific model is shown in Figure 1. To simplify the model, we made following assumptions:

(1) The population of the age group with the median represented each age group.
(2) The status of the chronic diseases of the population would not change during the simulation process.
(3) The hospitalization was the main result of the disease. Although the treatment of pneumococcal diseases included outpatient treatment and inpatient treatment in the process of the therapy, the condition of outpatient treatment was relatively mild, and there would be no death.
(4) The cost of missing work was not included in the cost as people aged 60 years or over generally had been in retirement in Shanghai, China.
(5) Each patient was accompanied by a family member during the illness.
(6) The cost caused by adverse reactions after vaccination was not considered, because the safety of PPSV-23 was reported to be good and the vaccination will only cause mild adverse reactions (17).
(7) Death of other causes was not considered after infection with pneumococcal related diseases.

### Cost and Resource Input

A Markov model was developed to estimate the costs and health outcomes with a 5% discount each annum based on the Chinese guidelines (18). The costs included the cost of buying vaccine, vaccination and management, patients' medical treatment and individuals who were unable to perform paid work because of hospitalization work for family members. The indirect cost was calculated by using the per capita daily wage in Shanghai and the average hospitalization days of patients. All model epidemiological and direct medical cost parameters were summarized in Table 1. Through expert consultation with 10 persons in charge of pneumococcal vaccination in the SCDC, four district CDCs and four community health care centers, the health care professionals' workloads in the vaccination process was calculated. At the beginning of the program in 2013 a total of 3,500 people who participated in the vaccination work devoted half of their work time to the PPSV-23 vaccination because of the hard promotion. In the later stage, the workload of vaccination personnel according to the half-day workload of a three-person working group for every 50 people vaccinated. The district CDC's workload is one person a year, and the community health care center's workload is 1/4 work time of 2.5 people per year. The annual income of each staff member is about $21, 312, and the data are shown in Table 1.

**Table 1:** The epidemiological, medical cost, and QALYs of the pneumococcal diseases.

**Parameters**	**Values**							
	**60–64**		**65–74**		**75–84**		**85+**	
	**Low**	**High**	**Low**	**High**	**Low**	**High**	**Low**	**High**
**CLINICAL DATA**								
**Incidence (cases/100,000 persons/year)**								
CAP^a^ (19)	288	723	1,299	1,437	5,031	4,188	5,031	4,188
Bacteremia (20)	2.7	13.4	3.8	14.4	4.4	13.5	4.6	12.7
Meningitis (20)	0.14	0.70	0.20	0.76	0.23	0.71	0.24	0.67
**Mortality (%)**								
CAP (21)	7.6	7.6	7.8	7.8	10.9	10.9	13.6	13.6
Bacteremia (20)	10.6	13.3	13.6	17.0	17.6	22.0	46.4	57.9
Meningitis (20)	10.6	13.3	13.6	17.0	18.1	22.6	50.3	62.9
**COST DATA**								
**Direct medical cost (US$)**								
CAP^a^ (19)	1,406	2,624	1,701	2,651	1,812	2,390	1,812	2,390
Bacteremia (19)	8,528	8,756	8,557	6,964	8,090	5,730	8,090	5,730
Meningitis (15)	10,825	10,825	10,825	10,825	10,825	10,825	10,825	10,825
**Indirect cost (US$)**								
PPSV-23 per dose^*^					27.64			
Vaccine administration^*^					35.64			
Indirect medical cost (22)					34.88/day			
**Duration of illness (days)**								
CAP days^*^					22.4			
Bacteremia days (7)					34			
Meningitis days (7)					34			
**QALYs**								
Without pneumococcal disease (7)	0.77	0.63	0.75	0.56	0.67	0.52	0.51	0.51
CAP (23)					0.2			
Bacteremia (23)					0.2			
Meningitis (23)					0.2			

*US $1 = Chinese Yuan 6.10*.

* *From local data*.

a *The definition of high-risk population in this study is people with chronic disease including hypertension, diabetes*.

### The Epidemiology of Pneumococcal Disease

Parameters for the epidemiological data were obtained from the published literature and through the expert consultation of nine epidemiologists, clinicians, and SCDC experts (15, 19–21).

### Vaccine Effectiveness

According to the relevant research, the PPSV-23 vaccine effect against CAP is 30.9%, and the protection period is 5 years (12). Previous studies have found that the protective effect of PPSV-23 against invasive pneumoniae diseases (IPD) including bacteremia and meningitis is 70% (13). The patients with diabetes are at high risk susceptible to pneumococcal diseases (24, 25). And this study used the proportion of people with diabetes in the total population to represent the proportion of people with chronic diseases. The prevalence rates of diabetes in people over 60 years old in China is 19.4% (26). The data of QALY for pneumococcal and no pneumococcal diseases was from published literature (7), and the data are shown in Table 1. The indirect loss is patients' family members monetary loss due to take care of inpatients missed working hours, which is calculated by the per capita daily wage and the average hospitalization days in Shanghai (7, 22), and the data are shown in Table 1.

The effectiveness was measured in quality-adjusted life year, and health system perspective was considered for the study. The incremental cost-effectiveness ratio (ICER) is the final indicator.

### Sensitive Analysis

A one-way sensitivity analysis was performed to test the robustness of our base-case findings, where each parameter was independently varied by ±25% from the base-case value, and to assess whether they have influenced the incremental cost-effectiveness ratio (ICER). The results of the one-way sensitivity analyses were presented in a Tornado diagram. Parameters included epidemiological parameters of pneumococcal diseases, cost of buying vaccine, vaccine management and vaccination, hospitalization costs of pneumococcal diseases, and so on. As there was no official cost-effectiveness threshold in China, three times per capita GDP of Shanghai in 2013 was used as the threshold. The per capita GDP of Shanghai in 2013 is $14,759, so the threshold was $44,277 (22).

## Results

### Base-Case Analysis

The results of the base case analysis are shown in Table 2. By the end of 2018, 1,091,967 people aged 60 years or over were vaccinated with PPSV-23. Comparing with no vaccination group, vaccination group cost $1,962,000 more and gained 10,321.3 QALY. The ICER was $190.1 per QALY, which meant comparing with the no vaccinated group, vaccinated group need spend $190.1 for every extra QALY. The subgroup analysis was done for different age and risk groups. The highest ICER in the aged 60–64 and low-risk group is 7,284.1, which was lower than the per capita GDP of Shanghai and much lower than the threshold of $44,277 in this study. In aged 65–74 and high-risk group and age group of 75–84 and above, the ICER is negative, which means compared with the no vaccination group, the people who are vaccinated with PPSV-23 will spend less cost and save more QALY. With the increase of age group and risk, the ICER will be away from the threshold. The ICER in high-risk groups were always lower than that in low-risk groups.

**Table 2:** Cost, QALYs and ICER in base case analysis.

**Age**	**Risk**	**Group**	**QALYs**		**Cost**		**Incremental cost effectiveness ratio ($)**
**(years)**					**($10,000)**		
60–64	Low	PPSV-23	3,378,056.0	2,085.5	28,191.9	1,519.1	7,284.1
		No vaccine	3,375,970.5	–	26,672.9	–	
	High	PPSV-23	633,853.9	616.4	8,166.5	128.9	2,090.6
		No vaccine	633,237.4	–	8,037.6	–	
65–74	Low	PPSV-23	2,315,566.7	2,727.9	32,417.7	680.9	2,496.0
		No vaccine	2,312,838.8	–	31,736.8	–	
	High	PPSV-23	436,387.3	600.5	8,414.5	−37.3	−621.9
		No vaccine	435,786.7	–	8,451.8	–	
75–84	Low	PPSV-23	611,975.8	3,298.5	12,918.8	−1,490.8	−4,519.5
		No vaccine	608,677.2	–	14,409.5	–	
	High	PPSV-23	125,591.0	588.9	3,182.7	−379.5	−6,444.1
		No vaccine	125,002.1	–	3,562.2	–	
85+	Low	PPSV-23	51,946.2	334.0	1,387.1	−179.2	−5,363.7
		No vaccine	51,612.2	–	1,566.3	–	
	High	PPSV-23	12,541.8	69.5	340.8	−45.8	−6,598.5
		No vaccine	12,472.3	–	386.6	–	
**Total**		PPSV-23	7,565,918.6	10,321.3	95,019.9	196.2	190.1
		No vaccine	7,555,597.4	–	94,823.7	–	

### Sensitivity Analysis

**Figure 2:**
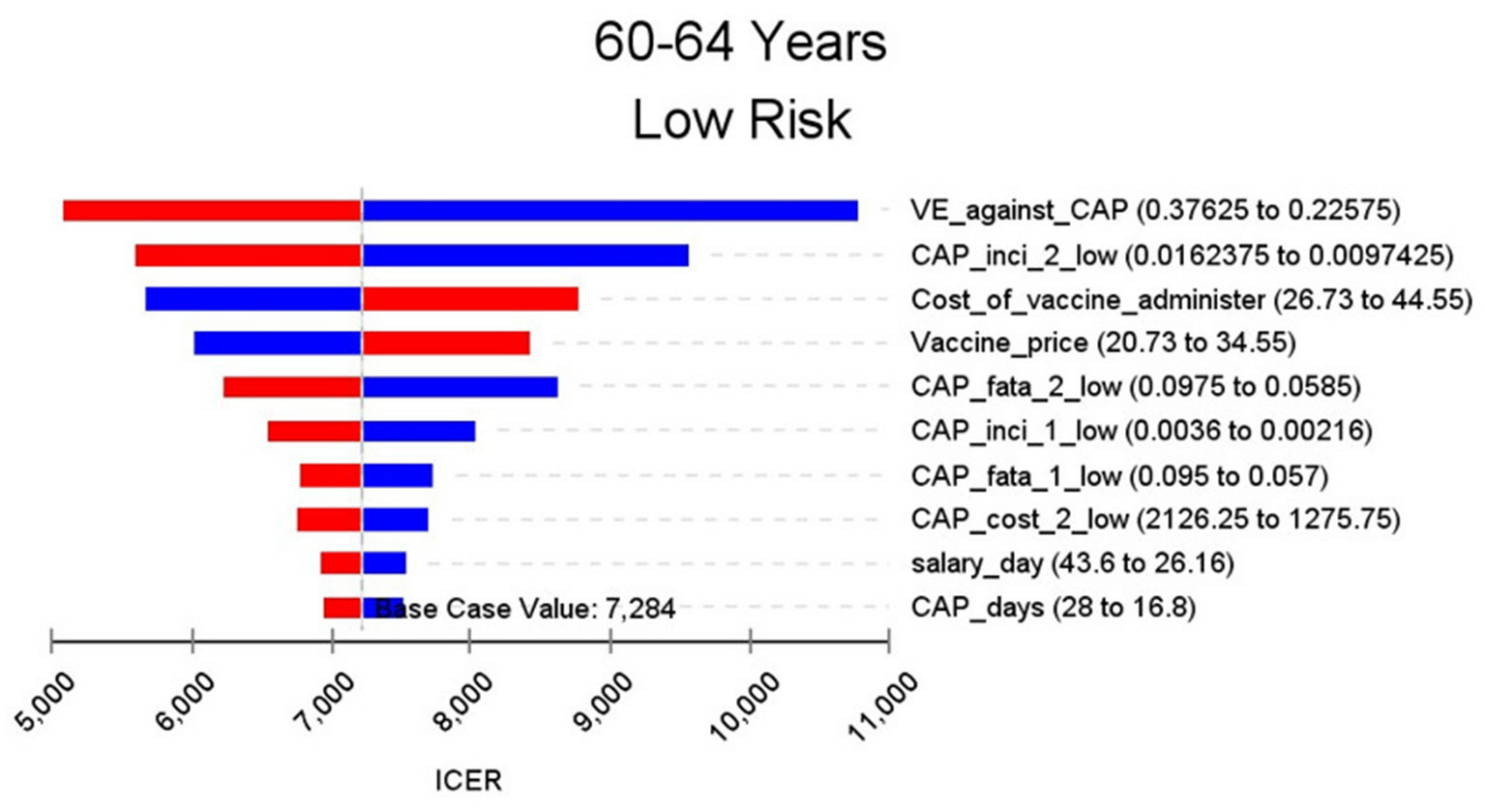
Sensitivity analysis in group 60–64 years and low risk.

**Figure 3:**
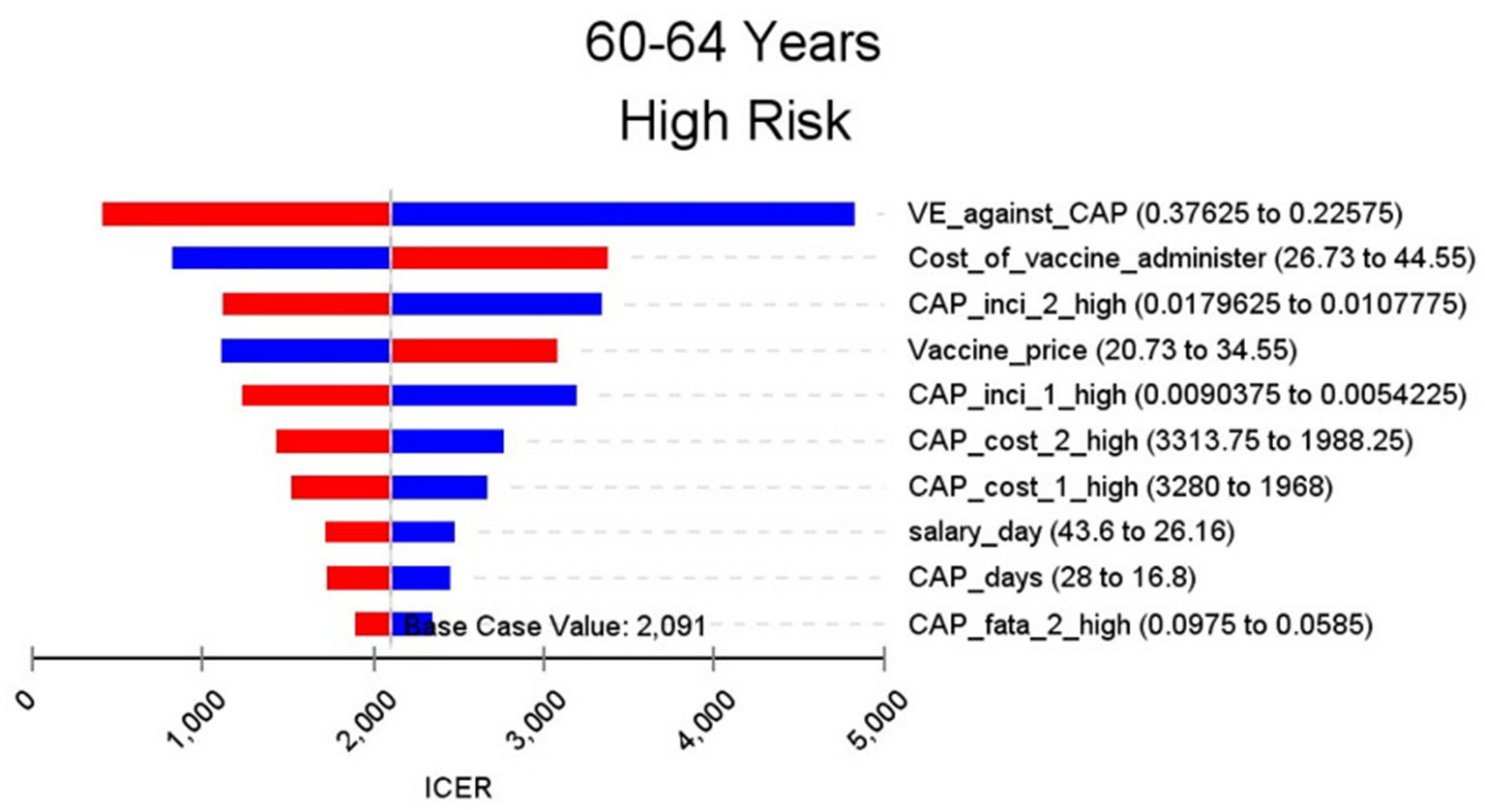
Sensitivity analysis in group 60–64 years and high risk.

**Figure 4:**
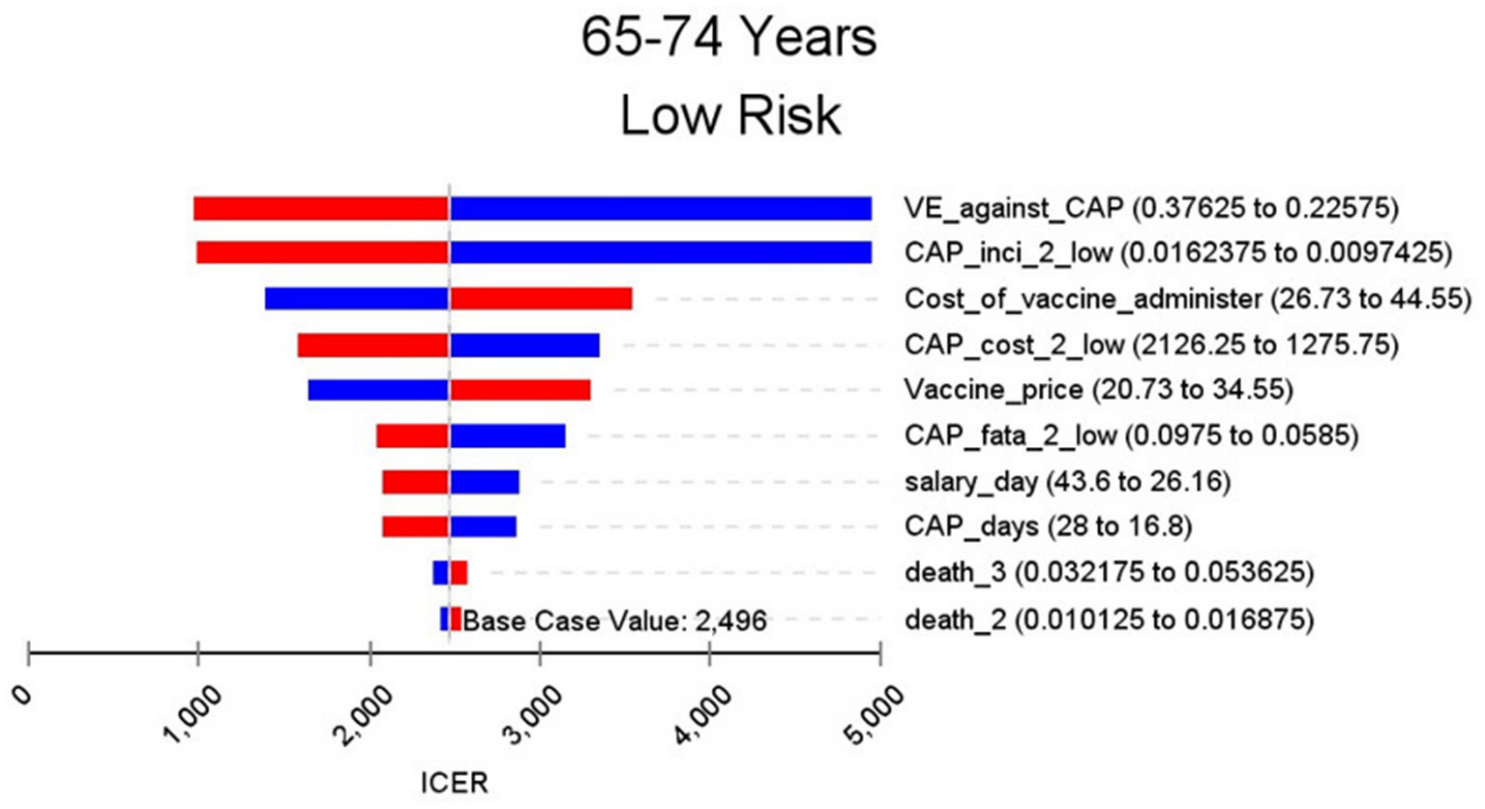
Sensitivity analysis in group 65–74 years and low risk.

**Figure 5:**
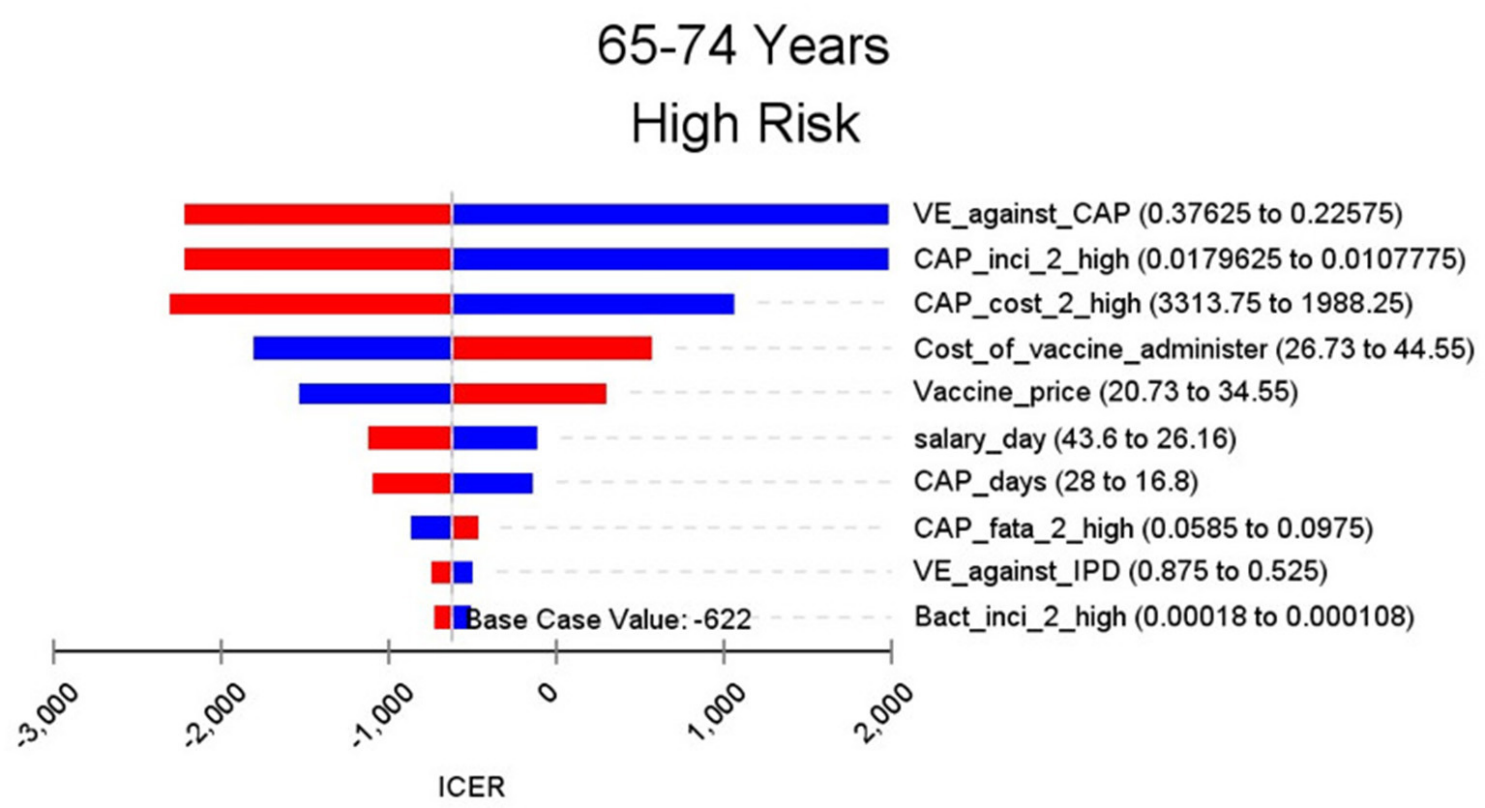
Sensitivity analysis in group 65–74 years and high risk.

**Figure 6:**
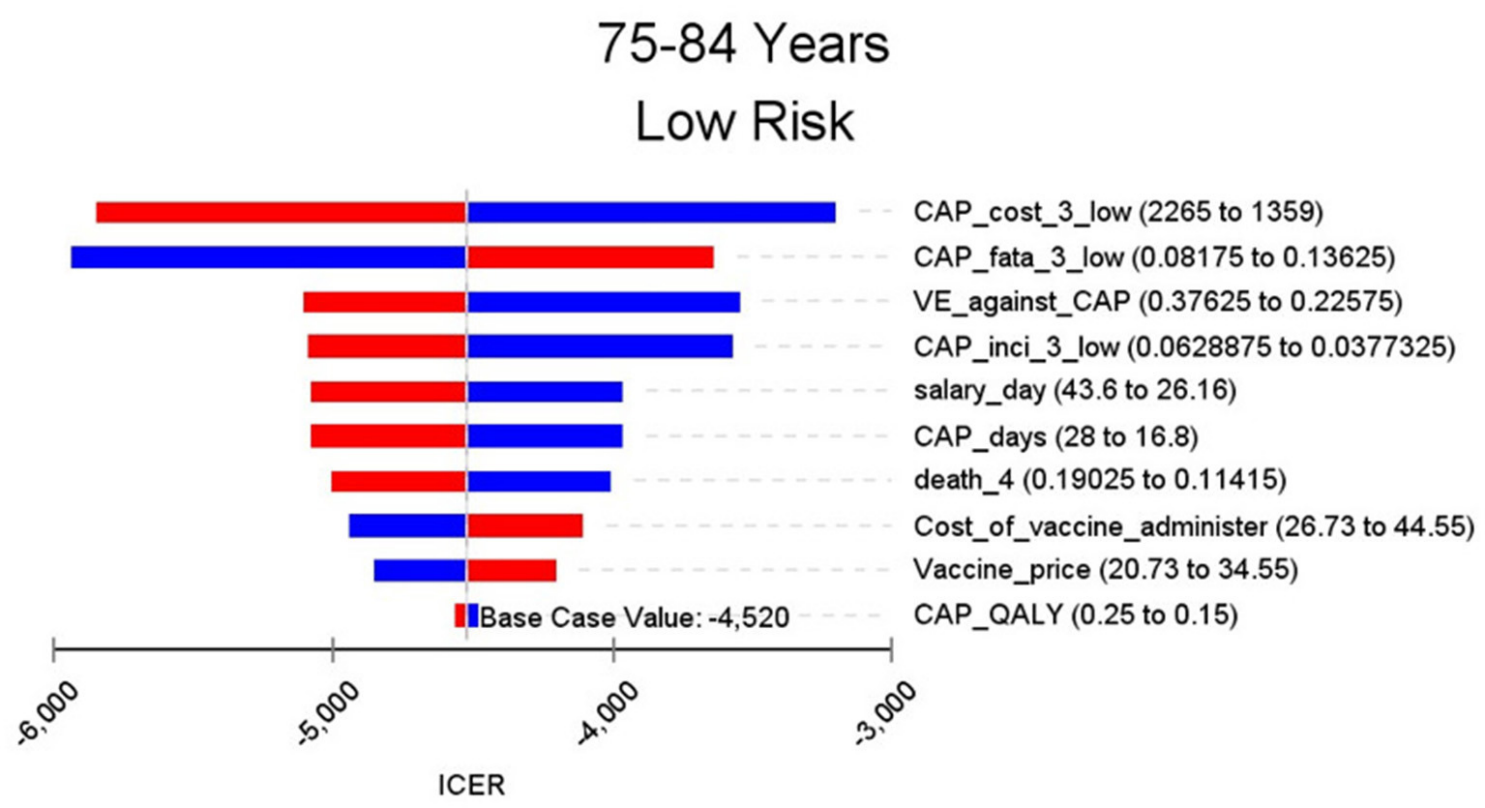
Sensitivity analysis in group 75–84 years and low risk.

**Figure 7:**
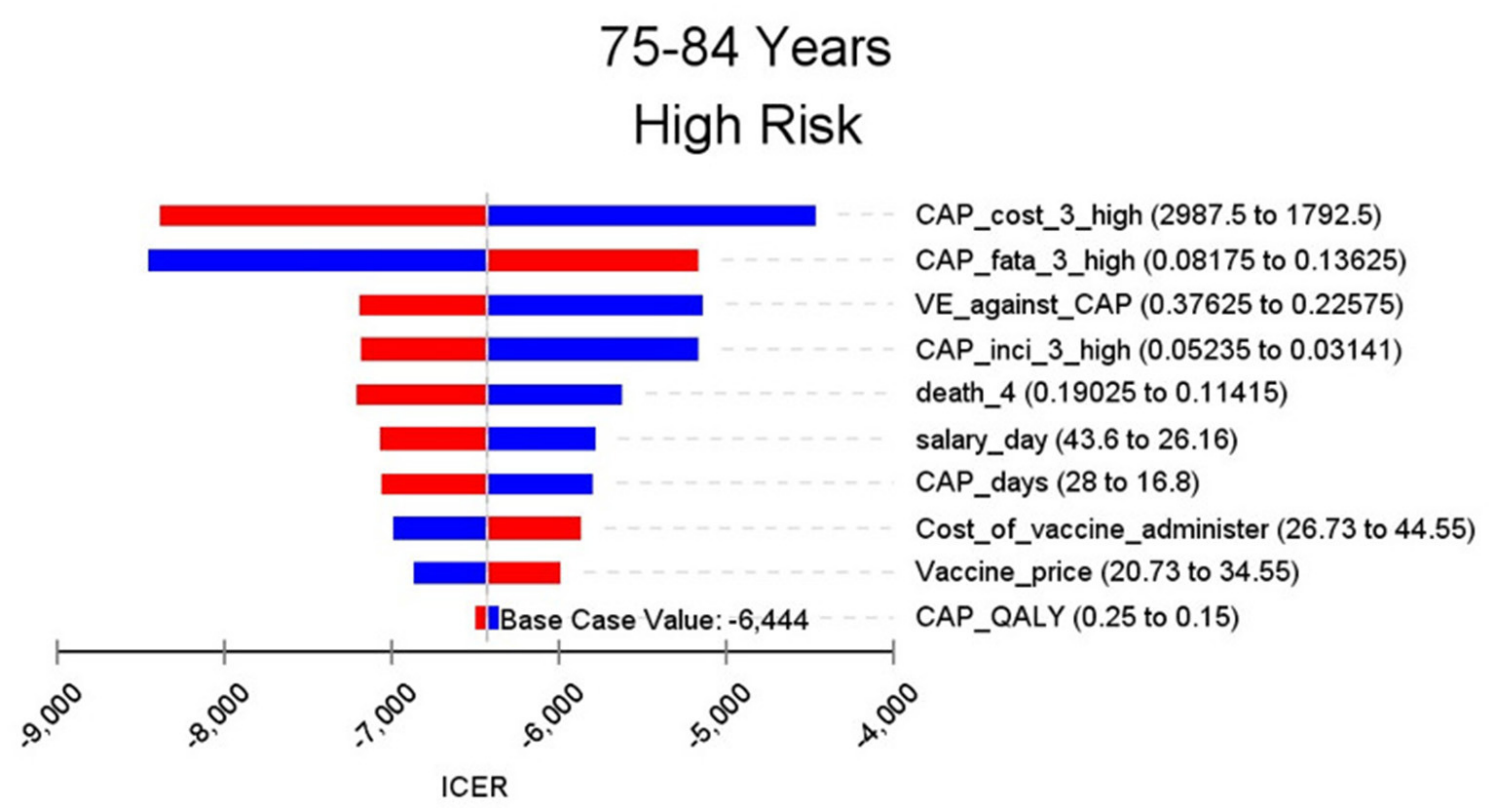
Sensitivity analysis in group 75–84 years and high risk.

**Figure 8:**
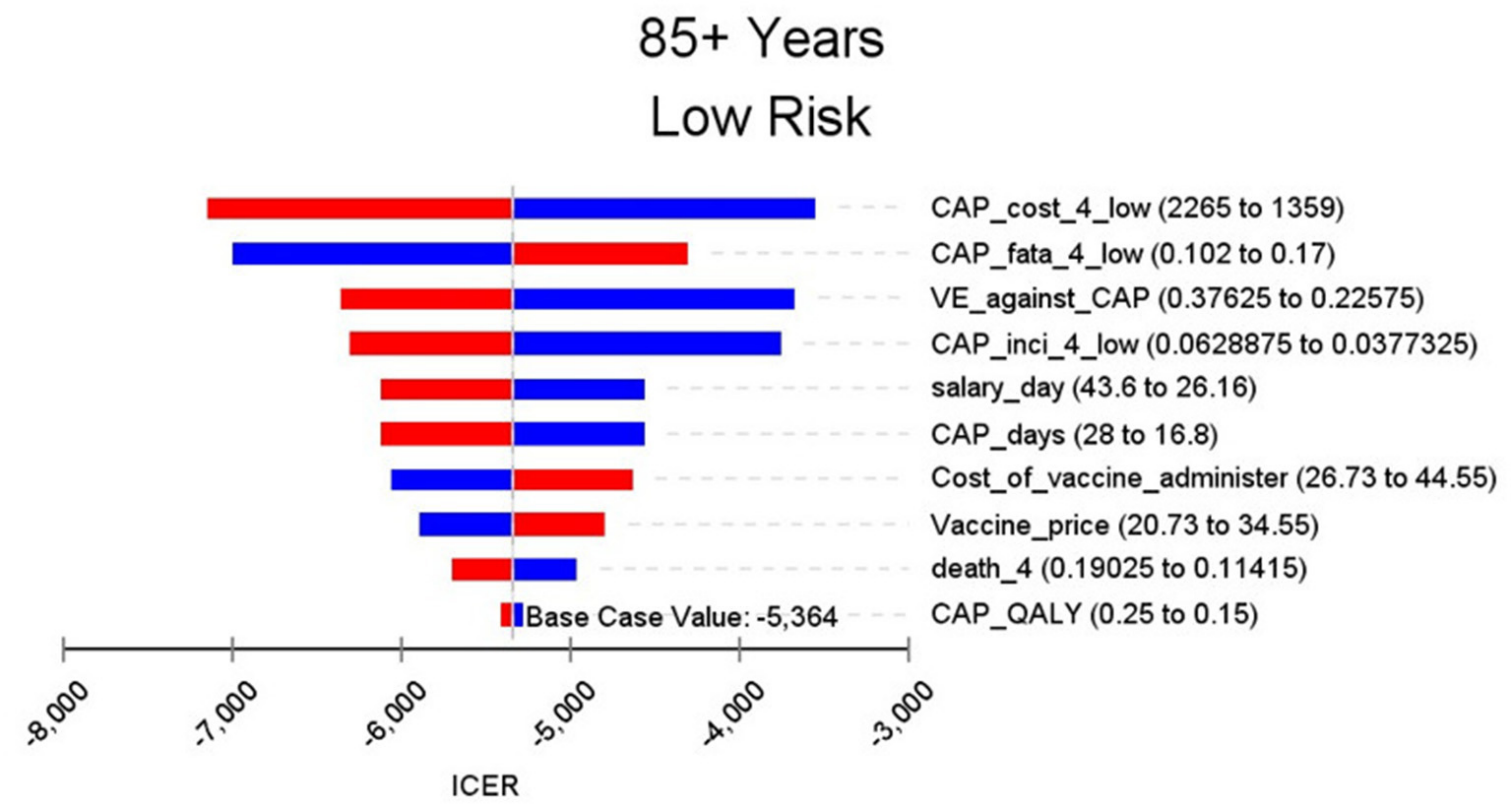
Sensitivity analysis in group 85+ years and low risk.

**Figure 9:**
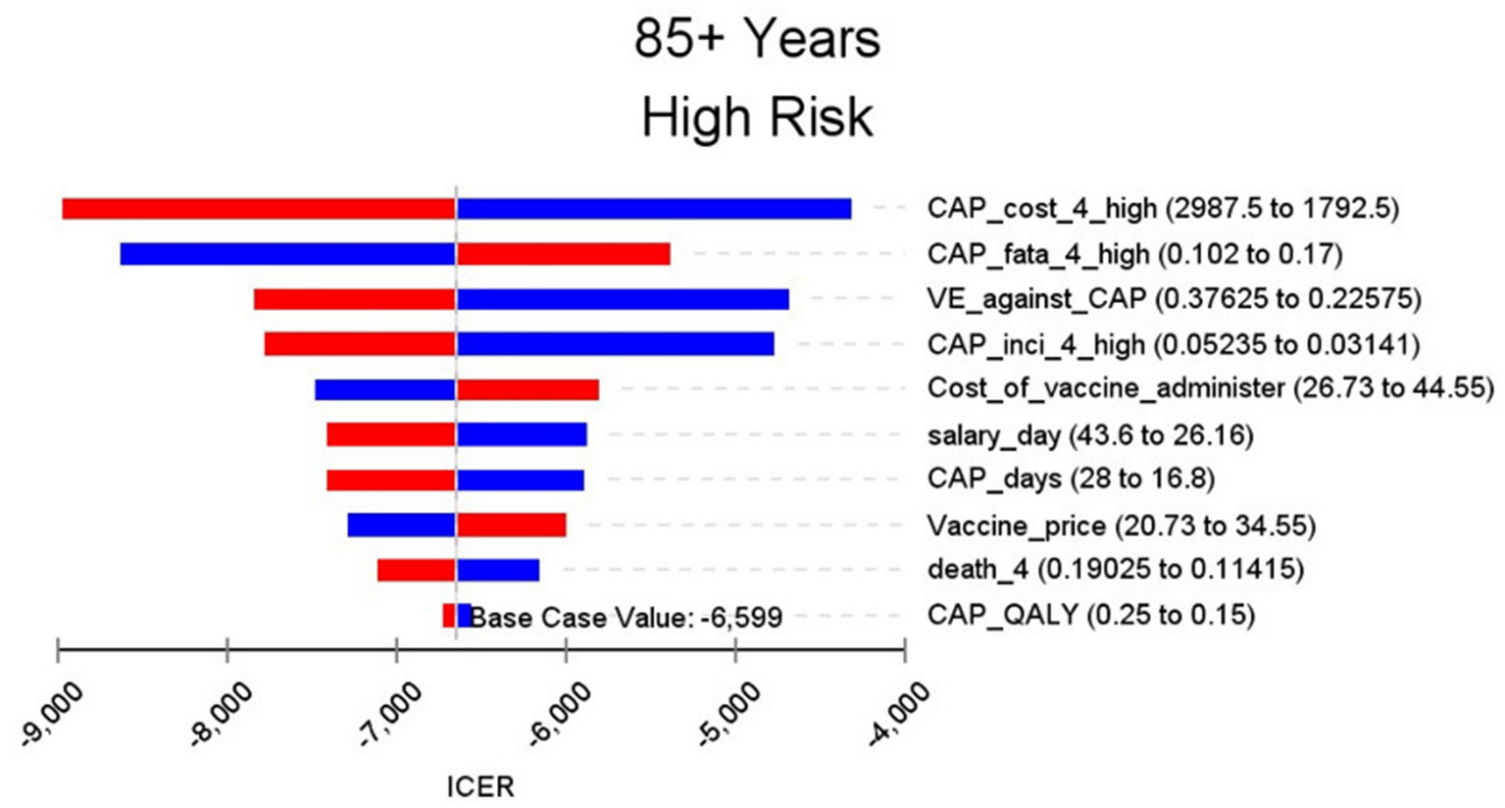
Sensitivity analysis in group 85+ years and high risk.

Figures 2–9 showed the results of the univariate sensitivity analysis of different age and risk groups. This figure contains the ten variables that have the most significant impact on ICER, most of which were PPSV-23 effectiveness against CAP, epidemiological data for CAP, and administrative costs for PPSV-23. The highest ICER was in the 60–64 low-risk group, about $11,000, when the PPSV-23 effectiveness against CAP reduces by 25%. It is far below the threshold levels used in this study.

## Discussion

The purpose of this study was to evaluate the economics of a major public health program in Shanghai that has vaccinated PPSV-23 for people 60 years or older for free since 2013. This study took the vaccinated population in the project as the target population, which was different from general economic evaluation research that was to simulate a group of people of the same age and the parameters change with the change of age (15, 19, 20). The study population was divided into eight different groups by age and risk.

Among all the eight groups, the ICER was lower than the per capita GDP of $14,759 of Shanghai in 2013 and significantly lower than the previously set threshold of three times the per capita GDP of $44,277 of Shanghai. It suggests that PPSV-23 vaccination is more cost-effective in the elderly aged 60 years or older compared to no vaccination. In the high-risk group, the benefit of PPSV-23 vaccination was more significant. Due to the chronic diseases, the incidence and mortality of pneumococcal disease in these high-risk groups are higher than those in the low-risk group. Compared with the low-risk group, the high-risk group could avoid more death and cases, vaccinating with PPSV-23 is more cost-effectiveness. Studies from UK (8), Japan (27), and Belgium (28) have also demonstrated that vaccination with PPSV-23 was a cost-effective intervention for preventing pneumococcal diseases. However, Chen et al. (29) suggest that compared with people who are not vaccinated, vaccination with PPSV-23 is not economical. It may be due to the study assumed that PPSV-23 has no protective effect on CAP, which is not consistent with Shanghai's actual situation.

Inconsistent with the results of previous studies, the several group's ICER of this study is <0. Previous studies set the same group of people to be vaccinated from 50 or 60 years old and simulate their situation to 100 years old. In these studies, the duration of protection after vaccination having been thought to last 5–10 years. If vaccinated at the age of 50 or 60, the protective effect lasts until 55–70. After that, the vaccine will no longer be effective. However, our includes the elderly over 70 years old who were vaccinated with PPSV-23. Furthermore, the morbidity, mortality, and treatment costs of pneumococcal diseases increased with age. The cost-effectiveness analysis of the subgroup showed that the ICER of PPSV-23 vaccination in the elderly over 75 years old and age in 65–74 high-risk group is <0. The costs of pneumococcal diseases avoided by vaccination PPSV-23 in these groups have exceeded the vaccination cost. Consequently PPSV-23 in the high age group may cost less and gain more QALY. One study has shown that influenza vaccination in 69-year-old people was economical (30). Pneumonia, influenza, and other lower respiratory infectious diseases are the only diseases that can be prevented by vaccines among the top 10 of the disease financial burden of the elderly in China (31). At present, the pneumococcal vaccine and influenza vaccine have not been included in China's immunization program. Chinese local data should be considered to use to make an economic evaluation of the two vaccines to help authorities to make relevant policies.

Through sensitivity analysis of eight subgroups, we found that the vaccine efficacy was a critical variable, leading to the largest variation in economic outcomes, which was also found by other published literature (20, 32). Besides, the epidemiological parameters of CAP and the cost of hospitalization have a great influence on the results in each group. In addition, vaccination's administrative cost is an essential factor affecting the ICER. At the beginning of the implementation of the major health services project in Shanghai, many resources were invested in the promotion and implementation of the project. As time goes by, the work for PPSV-23 is integrated into other routine work, and only little resources are needed to maintain this work. With the continuation of the project, the cost will be further reduced, and it will be more economical to vaccinate the elderly with PPSV-23.

## Limitation

There are several limitations to this study. Although most epidemiological and cost parameters were referenced based on the date outside the mainland of China, we still select the data source from the areas which are geographically similar with China. We use sensitivity analysis to test the stability of the model. However, care should be taken with interpreting these results. Our study makes some assumptions to simplify the model, such as not considering the loss of productivity of the elderly, only one family member accompanying the patient, which may be not completely same with but is consistent with the actual situation.

## Conclusion

The major public health service project in Shanghai to vaccinate people aged 60 years or older with PPSV-23 for free is economical, and its cost will be further reduced over time. More local epidemiological studies on pneumococcal diseases are needed to better evaluate the cost-effectiveness of PPSV-23 vaccination in China.

## Data Availability Statement

The original contributions generated for the study are included in the article/supplementary material, further inquiries can be directed to the corresponding author/s.

## Author Contributions

XS, XG, and HJ contributed to the conception and design of the study under the supervision of YL. JQ, XM, XG, YT, and HJ performed the data collection. YT and HJ conducted statistical analysis, wrote the manuscript, and revised as needed under the guidance of XS, XG, and YL. ZH and JR supervised the overall study and provided feedback throughout all stages. All authors contributed to the article and approved the submitted version.

## Conflict of Interest

The authors declare that the research was conducted in the absence of any commercial or financial relationships that could be construed as a potential conflict of interest.
